# Age-related Differences in Tongue-Palate Pressures for Strength and Swallowing Tasks

**DOI:** 10.1007/s00455-013-9469-6

**Published:** 2013-05-16

**Authors:** Tiffany Fei, Rebecca Cliffe Polacco, Sarah E. Hori, Sonja M. Molfenter, Melanie Peladeau-Pigeon, Clemence Tsang, Catriona M. Steele

**Affiliations:** 1 Swallowing Rehabilitation Research Laboratory, Toronto Rehabilitation Institute, University Health Network, 550 University Avenue, 12th Floor, Toronto, ON M5G 2A2 Canada; 2 Department of Speech-Language Pathology, University of Toronto, Toronto, ON Canada; 3 Institute of Biomaterials and Biomedical Engineering, University of Toronto, Toronto, ON Canada; 4 Rehabilitation Sciences, University of Toronto, Toronto, ON Canada; 5 Bloorview Research Institute, Holland Bloorview Kids Rehabilitation, Toronto, ON Canada

**Keywords:** Deglutition, Deglutition disorders, Tongue, Pressure, Aging

## Abstract

The tongue plays a key role in the generation of pressures for transporting liquids and foods through the mouth in swallowing. Recent studies suggest that there is an age-related decline in tongue strength in healthy adults. However, whether age-related changes occur in tongue pressures generated for the purpose of swallowing remains unclear. Prior literature in this regard does not clearly explore the influence of task on apparent age-related differences in tongue pressure amplitudes. Furthermore, differences attributable to variations across individuals in strength, independent of age, have not clearly been elucidated. In this study, our goal was to clarify whether older adults have reduced tongue**-**palate pressures during maximum isometric, saliva swallowing, and water swallowing tasks, while controlling for individual variations in strength. Data were collected from 40 healthy younger adults (under age 40) and 38 healthy mature adults (over age 60). As a group, the mature participants had significantly lower maximum isometric pressures (MIPs). Swallowing pressures differed significantly by task, with higher pressures seen in saliva swallows than in water swallows. Age-group differences were not seen in swallowing pressures. Consideration of MIP as a covariate in the analysis of swallowing pressures revealed significant correlations between strength and swallowing pressures in the older participant group. Age-group differences were evident only when strength was considered in the model, suggesting that apparent age-related differences are, in fact, explained by differences in strength, which tends to be lower in healthy older adults. Our results show no evidence of independent differences in swallowing pressures attributable to age.

The tongue plays an important physiological role in normal swallowing function. It is involved in the oral preparatory, oral transport, and pharyngeal stages of deglutition, contributing significantly to bolus formation, manipulation, and transport. Consequently, it is logical to think that impairments in tongue function (either limited strength or slowed movement) might lead to reduced tongue**-**palate pressure generation in swallowing, with possible functional consequences, including impaired bolus control and less efficient bolus transport. Several recent studies suggest that reductions in tongue strength with healthy aging may predispose seniors to swallowing impairment. However, due to the variety of instruments, methods, and tasks used to study tongue function, the extent to which age contributes to differences in tongue behaviours during swallowing remains unclear. The purpose of the study described in this article was to confirm the presence or absence of age-related changes in tongue**-**palate pressures generated during swallowing.

Both tongue strength and endurance are reported to decline in healthy aging, and the term *sarcopenia* has been used to describe age-related changes in muscle structure and function in the tongue [1–3], implying similarity to age-related changes seen in the limb musculature. One parameter that has been frequently used to study the effects of aging on the tongue is maximum isometric pressure (MIP), measured while a participant is pushing their tongue with maximum effort against the palate. Several studies have reported a pattern of declining MIP in healthy older adults [3–8]. However, these age-related changes appear to be seen only in certain tasks. Robbins et al. [3] measured lingual pressures using the Iowa oral performance instrument (IOPI) and concluded that although MIPs were reduced in healthy older adults, no statistically significant age differences were observed in peak pressures during saliva swallowing tasks. A more recent study by Youmans et al. [4] also used the IOPI and reported age-related reductions in MIPs and for swallowing tasks of honey-thick liquids and purees but not for thin or nectar-thick liquids.

Several studies in the literature provide convergent evidence that swallowing is a submaximal task with respect to its pressure requirements and that healthy older adults are capable of generating sufficient lingual pressures in this submaximal range during swallowing. Nicosia et al. [5] measured MIPs and bolus swallowing tasks using a three-bulb pressure array adhered to the palate and found that for both middle-aged and elderly men swallowing pressure amplitudes fell below 50 % of recorded MIPs. Robbins et al. [3] proposed a measure that captures the difference between tongue pressures generated in a MIP task and those generated in swallowing, which they called *functional reserve*. They argued that a reduction in functional reserve, attributable to a decline in MIP generation capacity (i.e., the upper end of the measure) constitutes a risk for swallowing impairment [3, 5].

Gender differences have also been studied with respect to tongue-palate pressure and its evolution with aging. An early study by Crow and Ship [6] concluded that men show a significant age-related decline in tongue strength but this effect, while significant, was not as marked in women. Utanohara et al. [7] recorded MIPs in more than 800 healthy adults. They reported significantly higher MIPs in men up until age 50 and a greater age-related decline in MIPs in men such that gender differences were not apparent in older participants. Stierwalt and Youmans [8] measured tongue-palate pressure capacity in healthy adults and adults with dysphagia using the IOPI. Significantly lower MIP values were found among their healthy control participants aged ≥60 compared to those <40 years of age and in women. However, a subsequent article by the same authors failed to find significant gender differences in MIPs; rather, women generated significantly higher mean pressures during swallowing tasks than men [4]. In a study of tongue-palate pressure modulation across different tasks in healthy adults under 40 years of age, Steele et al. [9] found higher pressures at the midpalate in women during bolus swallowing tasks, consistent with the findings of Youmans et al. [8].

One issue that has not been well controlled in prior studies of tongue-palate pressure generation is the possibility that measurements might vary within and across individuals as a function of slight differences in sensor placement. This dilemma is well recognized in data processing of physiological signals such as electromyography and is usually addressed by normalizing measures to a scale, such as a maximum effort task [10]. Tongue pressure measurement normalization using a maximum effort reference would clarify whether differences observed in tongue-palate pressure are attributable to differences in strength or other factors such as age, gender, or task. Although data normalization has not typically been used during the processing and analysis of tongue-palate pressure signals, Robbins et al. [3] explored the percentage of maximum pressure used during swallowing and found that young adults used a smaller percentage of maximum pressure than older participants. Youmans and Stierwalt [8] applied the same concept to their analysis and found that normalization neutralized significant differences in MIPs that were apparent between genders and age classes when using non-normalized data. The fact that sensors for tongue-palate pressure measurement differ in size, number, placement, method of force/pressure measurement (e.g., pneumatic vs. strain-gauge), and method of attachment (hand-held vs. glued to the palate or embedded in a pseudopalate prosthesis) [2–9, 11–16] provides further strong rationale for adopting a normalization convention to enable comparison across studies.

The goal of the current study was to clarify whether age, gender, and/or task differences exist in tongue-palate pressure amplitudes during swallowing using regular-effort saliva swallows (RESS) and water swallows as the tasks of interest. We were further interested to determine whether any age or sex differences in strength, measured during an anterior MIP reference task, influence the appearance of age, gender, and/or task differences in swallowing tasks, suggesting a need for data normalization in future studies.

We used path analysis, as illustrated in Fig. 1, to explore the following research questions:

**Fig. 1:**
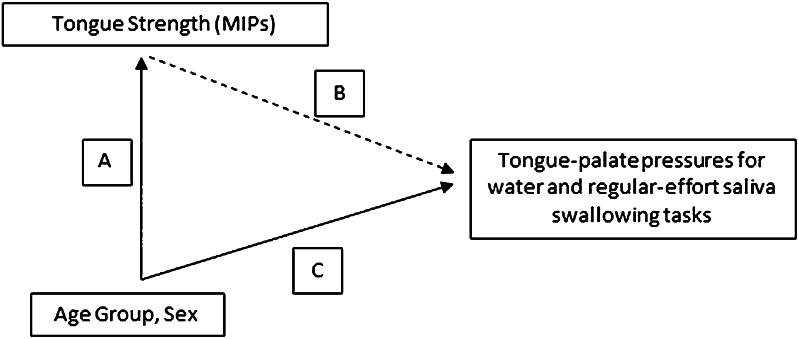
Path analysis used to explore the influence of strength, measured using a maximum isometric pressure task, on age group and sex differences in tongue-palate pressures during water and saliva swallows

1. *Path A*: does strength (i.e., tongue pressure amplitudes for MIP tasks) differ as a function of participant age and sex? Our hypothesis was that healthy older individuals of both sexes would display reduced MIP amplitudes compared to healthy younger controls.
2. *Path C*: do tongue-palate pressures for regular-effort saliva and water swallows differ as a function of task, participant age, and sex? Our hypothesis was that we would see differences in tongue pressures between saliva and water swallows, but we did not have a priori expectations about the directionality of these differences. We did not expect to see differences in swallowing pressures between age groups but we anticipated a possible age group × sex interaction, with higher pressures in younger male participants but no sex differences in older participants, based on prior literature.
3. *Path B*: provided that age and/or sex differences in MIP are confirmed in path A, do differences in strength modulate the task, age, and/or sex differences seen in regular-effort saliva and water swallows? The plan for this question was to use MIP as a covariate in a repetition of the path C analysis. Our hypothesis was that strength, as measured by MIP, would independently explain observed differences in tongue pressures used for swallowing, negating any apparent age or sex differences or interactions seen in path C.

An a priori power analysis showed that a target sample size of 80 participants would be needed to detect group mean differences of 18 mmHg attributable to the between-group factors of sex and/or age group with 80 % power.

## Materials and Methods

### Participants

Eighty-six healthy adults in two age groups (“young”: 18–40 years; “mature”: ≥60 years) consented to participate in the study, which was approved by the local institutional research ethics board. All participants passed an initial intake interview and reported no history of type I diabetes, chronic sinusitis, taste disturbance, or any swallowing, motor speech, gastroesophageal, or neurological difficulties. A list of medications known to influence saliva, level of consciousness, or swallowing was shown to all potential participants during intake, and those who disclosed regular use of these medications were excluded. Similarly, disclosed alcohol use of more than two drinks per week and smoking status within the prior 2 years resulted in exclusion. All participants passed a brief oral mechanism examination and water-swallow screening performed by a licensed speech-language pathologist before they were accepted into the study. Seven participants withdrew from the study and one participant was excluded due to difficulty following directions. The remaining 78 participants comprised a young age group of 21 women and 19 men with a mean age of 27 years (range = 18–39), and a mature participant group of 22 women and 16 men with a mean age of 70 years (range = 60–87).

### Data Collection

Tongue-palate pressure data were collected on the KayPENTAX Swallowing Signals Lab (KayPENTAX, Montvale, NJ, USA) using an array of three 13-mm-diameter air-filled bulbs, spaced 8 mm apart. The pressure bulb array was adhered to the midline of the participants’ palate using Stomahesive^®^ (Convatec, St-Laurent, Quebec, Canada), with the anterior bulb placed on the alveolar ridge, immediately behind the upper incisors. Tongue-palate pressures were recorded at a sampling rate of 250 Hz, with the bulbs calibrated to record an upper-amplitude limit of 750 mmHg. Data collection began with the maximum anterior isometric pressure reference task (MIP) and then proceeded to four additional tasks in one of four randomly assigned sequences. These tasks included RESS, effortful saliva swallows, maximum posterior isometric pressures, and water swallows. Each trial of a task involved a block of four task repetitions performed at a comfortable rate selected by the participant. Only the data for the MIP, RESS, and water swallows are discussed in this article.

### Data Processing

**Fig. 2:**
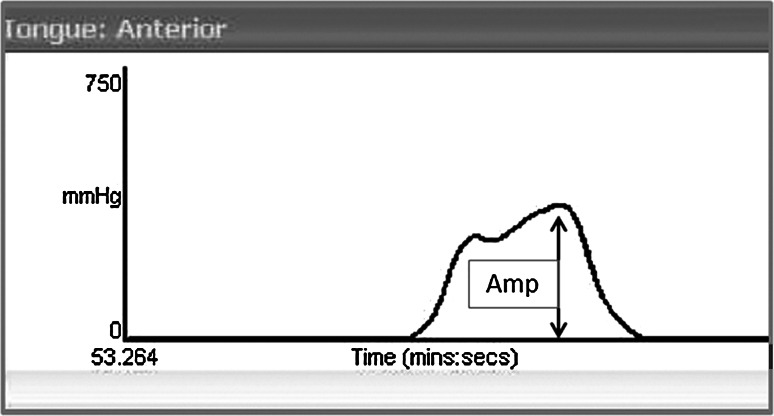
Illustration of a tongue-pressure waveform collected at the anterior palate, showing how amplitude was measured

Pressure waveforms for each bulb (anterior, medial, and posterior) were displayed on a computer monitor. The boundaries of a time envelope containing the onsets, peaks, and offsets of each task repetition were identified by two trained research assistants using a cursor function and documented. A custom MatLab algorithm (MathWorks, Natick, MA, USA) was then used to objectively identify minimum and maximum pressure values for each sensor waveform within each task time envelope. The pressure envelope was defined as the interval between the earliest onset of pressure and the terminal offset of pressure (i.e., return to zero) across the three sensors. Pressure amplitude (in mmHg) was calculated as the amplitude difference (in mmHg) between the lowest baseline pressure (typically zero mmHg) and the highest peak pressure seen across all three pressure waveforms for a given pressure task repetition. Figure 2 provides an illustration of the pressure amplitude parameter for a single swallowing event recorded at the anterior palate sensor.

### Analysis

Statistical analyses were performed using SPSS 21.0 (IBM, Armonk, NY, USA) using an alpha criterion of *p* < 0.05. Path A involved a linear mixed-model analysis of variance (ANOVA) with repeated measures, which was used to explore differences in MIP with factors of age group (young; mature) and sex, and a repeated factor of task repetition. For path C, we conducted a linear mixed-model ANOVA with repeated measures for the parameter of pressure amplitude with factors of TASK (RESS, WATER), age group (young; mature), and sex, with post hoc Sidak tests to identify significant pairwise comparisons. Task repetition nested within trial was used as the repeated-measures factor. Model simplification was then pursued systematically by removing sex from the model and exploring age-group differences in swallowing pressure amplitudes within task. Effect sizes were calculated using Cohen’s *d*, which examines group mean differences as a function of pooled standard deviation [17]. According to this measure, when *d* = 0.5–0.7, the effect can be considered moderate, while values of *d* > 0.7 are considered strong. Cohen’s *d* values <0.5 are considered weak.

## Results

**Fig. 3:**
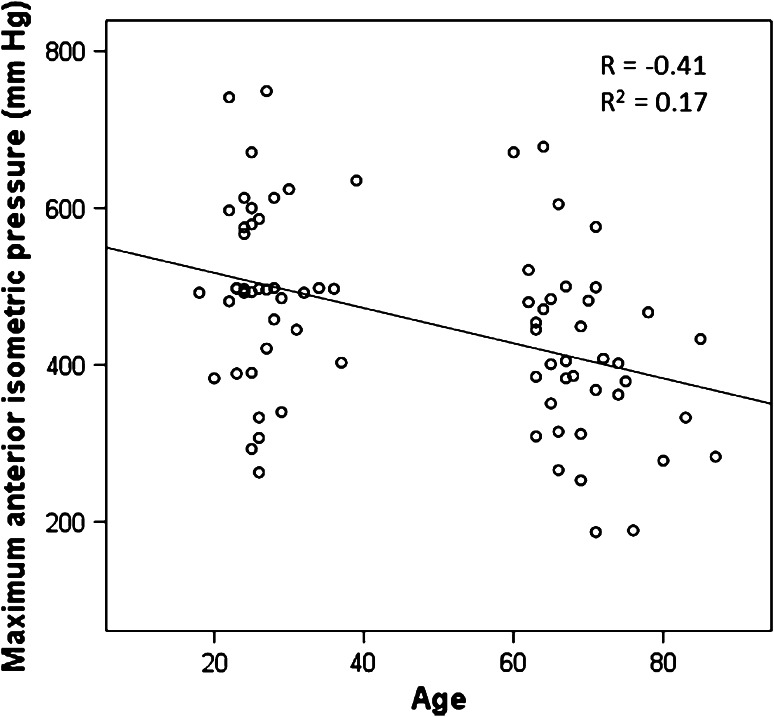
Relationship between tongue strength, measured during a maximum isometric pressure task, and age

Descriptive statistics for pressure amplitude are given by age group, sex, and task in Table 1. Significantly higher MIPs were found in the younger participant group, as shown in Fig. 2 [*F*(1, 73.05) = 18.45, *p* = 0.000], with a strong effect size of Cohen’s *d* = 0.99. There were no significant differences in MIP between male and female participants and no significant age group × sex interaction. Examination of the distribution of strength scores by age group, with age as a continuous parameter, showed a statistically significant negative correlation (*R* = −0.41) between strength and age, but also considerable overlap between groups, such that this sample included both younger individuals with relatively weak MIP measures and older individuals with relatively strong MIPs (see Fig. 3).

**Table 1:** Descriptive statistics for tongue-palate pressures (in mmHg) shown by participant age group, sex, and task

Task	Age group	Sex	Mean	95 % CI		SD
				Lower boundary	Upper boundary	
Maximum anterior isometric pressures (MIPs)	Young (<40)	Female	422	395	448	122
		Male	486	463	509	100
	Mature (>60)	Female	343	311	376	149
		Male	322	277	367	179
Regular effort saliva swallows (RESS)	Young (<40)	Female	120	105	135	69
		Male	158	133	183	109
	Mature (>60)	Female	121	110	133	54
		Male	118	104	132	56
Water swallows	Young (<40)	Female	88	77	98	48
		Male	124	102	145	93
	Mature (>60)	Female	89	81	98	39
		Male	90	77	104	53

**Fig. 4:**
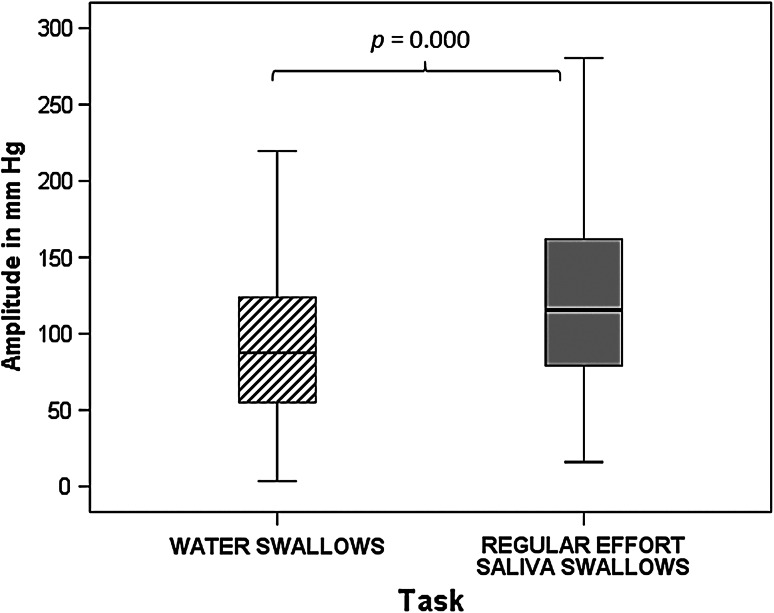
Differences in anterior tongue-palate pressure amplitudes for water-swallowing and regular-effort saliva-swallowing tasks

The initial results of the path C model revealed statistically significant differences in swallowing pressure amplitudes as a function of task, with higher pressures seen in RESS than in water swallows [*F*(1, 531.58) = 54.46, *p* = 0.000]. Cohen’s *d* revealed only a weak effect size (*d* = 0.26) for this task difference, which is illustrated in Fig. 4. No other factors were significant at this level of the model. Consequently, subsequent analyses were performed within task to determine whether age-group or sex differences existed within saliva swallows or water swallows, respectively. No significant differences according to either factor were found.

**Fig. 5:**
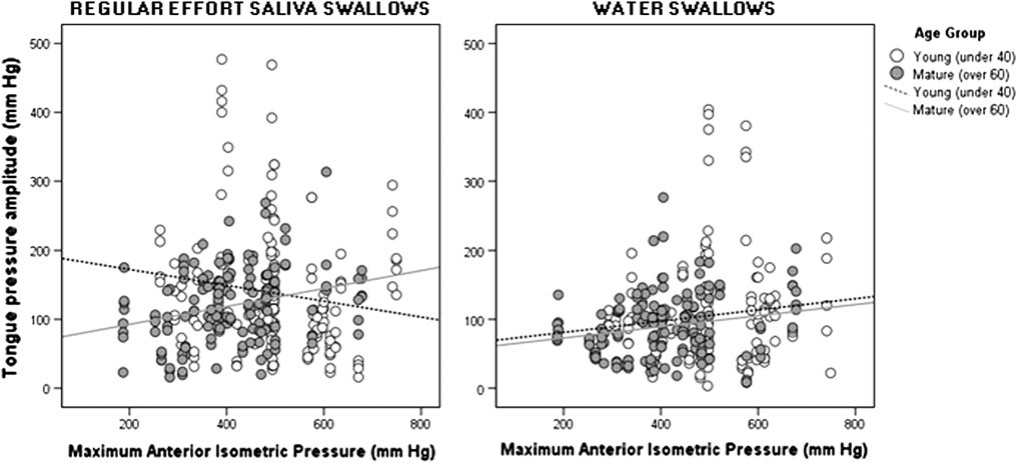
The three-way interaction between age group, task, and tongue strength seen for swallowing pressures

Turning to path B, our interest was first to determine whether consideration of MIP as a covariate would influence the results of the path C models. Given evidence that sex did not influence MIP or the initial path C results, sex was excluded from the path C model. A statistically significant main effect of task [*F*(1 533.43) = 17.456, *p* = 0000] with significant two-way task × age group [*F*(1, 533.43) = 12.684, *p* = 0.000] and task × MIP [*F*(1, 533.66 = 4.612, *p* = 0.032] interactions and a significant three-way task × age group × MIP interaction [*F*(1, 533.66 = 11.540, *p* = 0.001] was found. Further inspection of the three-way interaction, as shown in Fig. 5, revealed a nonsignificant trend of declining saliva swallow pressures with increasing MIP in the younger participants (*R* = −0.14, *R* ^2^ = 0.02), while older participants with higher MIPs showed the opposite trend of increasing saliva swallow pressures (*R* = 0.28, *p* = 0.001, *R* ^2^ = 0.08). By contrast, in the water-swallowing task, participants in both age groups showed trends of increasing swallow pressures with increasing MIP (young: *R* = 0.12, *R* ^2^ = 0.014; mature: *R* = 0.20, *p* = 0.014, *R* ^2^ = 0.04). Thus, in the older participants, a pattern of increasing pressures was seen for both saliva- and water-swallowing tasks as MIP increased.

## Discussion

This study adds to our understanding of tongue pressure by bridging gaps in prior literature. Specifically, Robbins et al. [3] previously reported data for maximum isometric pressures and saliva-swallowing pressures collected using the IOPI, which involves a single hand-held pneumatic bulb sensor of approximately 2.7-ml volume. In later studies, Robbins et al. [2] recorded maximum isometric pressures and bolus swallowing pressures using the KayPENTAX tongue bulb array system, which involves a series of three small pneumatic bulbs that are glued to the palate. Youmans et al. [4, 8] used the IOPI to explore maximum isometric pressures and bolus-swallowing pressures with liquids of different consistency. The current study is, to our knowledge, the first to describe maximum isometric pressures, saliva-swallowing pressures, and bolus-swallowing pressures (water) together, all measured with the same instrument (the KayPENTAX system). By doing so, our study brings task differences in tongue-palate pressures into clearer focus, and suggests that task differences may have been confusing the picture historically with respect to the question of whether swallowing pressures decline with age. The data confirm and clarify that neither saliva- nor water-swallowing pressures decline as a function of healthy aging. At the same time, the data also corroborate previous evidence that maximum isometric pressures are lower in healthy older adults when compared in a groupwise fashion to those of healthy younger adults.

Given that tongue strength on MIP tasks is reduced in older participants but declines are not seen in swallowing pressures, the remaining question is whether individual differences in strength, measured in the form of tongue pressure generation capacity, influence (or limit) the amplitude of pressures that are generated during swallowing. Previous studies have arrived at conflicting conclusions regarding this question, based on decisions about whether to express measures of functional reserve in percentage units, normalized to an individual’s MIP measures [2, 4, 5]. In this study, we used path analysis, incorporating the influence of strength as measured by MIP as a covariate in analyses of age-group differences in swallowing pressures. The results show that age-group differences in saliva- and water-swallowing tasks are evident only when strength is considered. Given that the distribution of strength in our sample differed by age group, this finding strongly suggests that strength, rather than age, influences the pressures used in swallowing. Furthermore, we found that older participants generate both water and saliva swallows that are significantly correlated with their tongue strength, as measured in the MIP task. Given the fact that our sample included older people with relatively strong maximum isometric pressures and younger people with relatively weak maximum isometric pressures, our study suggests that strength rather than age is a better explanation of the differences in swallowing pressures seen in this study sample.

Evidence that bolus-swallowing pressures appear to be correlated with measures of tongue strength provides a strong argument for normalizing measures of tongue pressure against a context of MIP in future research, or, alternatively, for incorporating strength measures as covariates in analysis. Controlling for individual or groupwise variations in strength in this manner will help to bring variations attributable to other experimental factors like age and sex into clearer view, should they exist. Such measures will also permit the comparison of data across instruments, which is a consideration of increasing importance as the number of instruments for measuring tongue pressures grows in number and design.

## Conclusions

In conclusion, this study addresses a continuing debate in the literature regarding the presence of declining tongue strength for swallowing tasks with age. Our study shows that the tongue pressures required for swallowing differ in amplitude by task. Individual differences in tongue-pressure generation capacity do influence the pressures generated in bolus-swallowing tasks with water in healthy older participants. This influence is not seen in healthy younger adults and is not as strong in saliva swallows. This finding suggests the interesting possibility that boluses of increased viscosity that require greater propulsive forces may elicit swallow pressures that are increasingly dependent on strength. Certainly, data from recent studies of swallowing pressures with different types of boluses support this suggestion [4, 16]. Thus, we conclude that age alone does not constitute a reason to expect reduced swallowing pressures for water- or saliva-swallowing tasks in seniors.
